# Supplementary material for: The effect of the choice of screening test when measuring the prevalence of gambling disorder: A cross-sectional study in Japan
Source: PLoS One. 2025 Apr 1;20(4):e0318885. doi: 10.1371/journal.pone.0318885 (PMC11960946; doi:10.1371/journal.pone.0318885)
Supplement: Attachment — Submitted filename: Response_to_Reviewers.docx [file pone.0318885.s001.docx]

**Responses to comments from Reviewers**

**Comments from Reviewer #1:**

We appreciate your thoughtful peer review of our paper. Many of your points align with our perspective, and your insights were invaluable. We have addressed your comments as detailed below and revised the main manuscript accordingly. As part of the in-depth discussions held during this revision, we added one author and redefined the roles of the authors.

**Reviewer #1:Summary**

There is a lot of interesting evidence here, and the dango chart seems useful for comparing psychometric tools. However, I think the narrative of this paper could be developed more. I find myself asking what the main message is and how it informs the field at large. What stands out to me looking at this data is how limited the functionality of gambling screening tools is in that they use largely arbitrary cut-offs to identify risk, as exemplified by the sometimes-drastic differences across the present data. I think there is a reasonable argument to be made that they do not identify the totality of harm and instead focus on those regarded as high risk. From my perspective, the findings tell a meaningful story, but I am not sure that story is discussed as well as it could be. In some areas the narrative and supporting evidence feels rather old and outdated. I have included some papers in my feedback that the authors might find useful.

Broadly speaking, I believe (and the evidence suggests) that gambling screening tools are useful to a point but are inherently flawed as they are derived from a clinical perspective of harm. This leads to them only measuring those with significant issues (even tools like the PGSI). This leads to low prevalence rates, giving the impression of a niche issue. However, harm exists on a continuum and those who sit lower down (low risk, medium risk) are often not accounted for or missed by such tools as they measure expressions of harm and not things predictive of risk. As such, I believe any discussion of the gambling prevalence data needs to have this limitation in mind and should be discussed as part of the work's narrative. If the long-term aim is to track the changes in harm in these two areas as a result of the casino, then the simple application of the SOGS, PGSI (or others CPGI, GABs, NODS, etc.) is not enough to track the changes in risk on a societal level, and a more holistic approach that takes into account risk factors is needed. This point should be made clear in the paper.

I recognise that the authors may not wish to make such an amendment, but it could lead to a paper that meaningfully contributes to the literature.

**Response:**

Thank you for your comments.

Thank you for your comments. Although we are not entirely certain that we have fully captured your perspective, we agree with your observations. It is indeed important to adopt a comprehensive public health approach to gambling disorders, similar to the SBIRT model used for alcohol use disorders. Specifically, we believe it is essential to understand not only the prevalence of addiction among high-risk groups but also long-term trends in medium- and low-risk groups. The Dango chart in our study illustrates a continuous distribution of scores. We believe that clarifying the risks among participants below the cutoff score represents a crucial area for future research. While this paper was not designed from that perspective, we have revised the discussion section to address this issue. For details, please refer to our response to Comment D below.

**Comment:A**

Line(s) 32: Its not clear why the lie bet was used. it's accuracy and reliability are fairly underwhelming. its only befit is its lengths but there are other tools (like the brief biosocial gambling screen) which are short and more psychometrically robust.

**Response:**

The Lie/Bet questionnaire was employed in this study because it is a condensed iteration of a screening instrument used in a Japanese survey on actual conditions . While the study by Dowling, Merkouris, Rodda, Manning, Youssef, Lubman, and Volberg (2019), which you recommended, demonstrates a certain level of validity, it is unlikely to gain widespread use as a screening tool in Japan. Therefore, we believe it should not be included in future studies.

**Comment:B**

Line(s) 52- 54: Is this context needed here? Feels out of place.

**Response:**

PLOS ONE is a multidisciplinary research journal that caters to readers with diverse backgrounds, including varying levels of familiarity with gambling behavior. To ensure the topic is accessible, we included background information in the manuscript.

**Comment:C**

Line(s) 57- 58 & 61 - 63: This source is 12 years old. Given that you are citing prevalence data it should be the most up to date statistics.

**Response:**

We are grateful for your recommendation and have replaced the cited reference with the most recent literature. However, as the increase or decrease in prevalence is mentioned in [4], that portion of the quotation has been retained.

**Revised text:**

**Line 54-56:**

The prevalence of problem gambling in the past 12 months varies widely depending on the screening test used, ranging from 0.12% to 5.8% [4].

**Line 59-66:**

However, the prevalence has been declining since the late 1990s in the United States and the early 2000s in Australia [5]. Conversely, the prevalence of problem gambling increased in certain countries and regions during the early to late 2000s. Changes in the gambling environment, including the expansion of internet gambling, are thought to be contributing factors to this increase [4]. In Japan, results from the SOGS-R indicate a prevalence of problem gambling of 8.04% [6]. Nevertheless, no systematic fact-finding surveys have been conducted in Japan to determine whether the prevalence of possible gambling disorder has increased or decreased.

**Comment:D**

Line(s) 78 – 80: I'm not sure I agree. Yes, this would lead to a more complete summary of the prevalence but what would that actually tell you? The key issues with population survey are that they do not help inform harm prevention because the majority of the cases (low risk gamblers) are not identified in the data. In other words, the prevalence only represents the tip of the iceberg. Some commentary on this limitation would be useful.

**Response:**

In our manuscript, we focused on the cut-off score for each screening test (possible gambling disorder). However, the continuous score distribution of the Dango chart provides additional insights. It is possible that the group below the cut-off score includes people who may develop gambling-related harm in the future. We also believe a preventive approach is necessary for gambling disorders, and we consider the SBIRT model (screening, brief intervention, and referral to treatment) for alcohol use disorders tobe applicable in this context. We have incorporated this perspective into the Discussion section.

**Revised text:**

**Line 209-219:**

Although respondents scoring below the cut-off in each test have not received substantial attention, the continuous score distribution in the Dango Chart offers insights into their actual situations. It is plausible that this group includes individuals who may develop gambling-related harm in the future. Regarding alcohol use disorders, the screening, brief intervention, and referral to treatment (SBIRT) approach is widely used as a preventive and comprehensive public health strategy. Adapting SBIRT's concepts and methodologies to gambling disorders could be beneficial [34]. In alcohol use disorders, appropriate screening enables suitable interventions; a similar prevention-focused approach would be valuable for gambling disorders [35]. To manage the spectrum of gambling-related harm to pathological gambling, long-term monitoring and continuous evaluation methods, such as the Dango Chart, would be advantageous.

**Comment:E**

Line(s) 83: It's also worth noting that gambling screening tools (even the SOGS) are not measuring gambling disorder, they are simply counting symptoms presumed to be indicative of it. As such its more appropriate to discuss this by using the term gambling related harm. Unless there is a diagnosis form a clinician guided by the DSM (or ICD) then the term gambling related harm is more fitting.

Line(s)86: Try to draw on a consistent terminology. Gambling addiction is not listed in the ICD or the DSM. Again, the term gambling related harm is a better umbrella term.

Line(s) 131: Gambling related harm

Line(s) 177: amend to “those experiencing gambling related harm”

**Response:**

Thank you for this observation. In this study, we used the term "gambling disorder" to refer to participants exceeding the cut-off point for each screening test. This is, however, not equivalent to a confirmed diagnosis. We have revised the terminology to "possible gambling disorder” and included the term “gambling related harm” in the Discussion to describe individuals with problematic gambling behaviors regardless of their score. In the Introduction, we have used the terminology as presented in the original cited research.

**Revised text:**

**Line 37-38:** The SOGS screens more individuals with a possible gambling disorder than other measures,

**Line 54-55**: The prevalence of problem gambling in the past 12 months

**Lines 56-57:** the prevalence of problem gambling began increasing

**Lines 60-62:** Conversely, the prevalence of problem gambling increased in certain countries and regions during the early to late 2000s

**Lines 63-64:** results from the SOGS-R indicate a prevalence of problem gambling of 8.04%

**Lines 65-66:** prevalence of possible gambling disorder.

**Lines 88-89:** cut-off score on the prevalence of possible gambling disorders.

**Line 136:** Table 3 shows the prevalence of possible gambling disorders based on

**Line 149:** The prevalence of possible gambling disorders

**Line 151:** Regarding the total number of people with a possible gambling disorder by region,

**Line 171:** in surveys on the prevalence of possible gambling disorders

**Line 174:** on the prevalence of possible gambling disorder

**Line 180:** suffer from a possible gambling disorder than women;

**Line 182-183:** the differences in possible gambling disorder prevalence by sex.

**Line 186:** Second, when comparing the distribution of prevalence of possible gambling disorder

**Line 241:** screening tests for possible gambling disorders

**Line 243:** when measuring the prevalence of possible gambling disorders.

**Line 245-246:** significantly impacts the prevalence of possible gambling disorders.

**Line 251:** surveying the prevalence of possible gambling disorders.

**Comment:F**

Line(s) 96: Why was Fukuoka selected as a point of comparison? Some justification (even something minor) would be useful.

**Response:**

Thank you for this observation. Fukuoka was selected as a city without planned casino developments. However, as noted in Comment O, we have shifted our focus in this revision from analyzing the impact of casino openings to evaluating the scales themselves. Therefore, the reasons for selecting the cities are now mentioned briefly.

**Revised text:**

**Line 92-94:**

We conducted an online survey in two Japanese prefectures with comparable population sizes: Osaka, where an integrated resort including casinos is scheduled to open in 2029 [24], and Fukuoka, where no such resort is planned.

**Comment:G**

Line(s) 102 – 103: The PGSI and the Lie bet are not measuring gambling disorder. Amend to gambling related harm.

**Response:**

Thank you for your observation. We have added a detailed explanation of the screening tests to the Introduction and Materials and Methods section, considering Reviewer 2's comments (M and P).

**Revised text:**

**Lines 68-74:**

The first of these is the South Oaks Gambling Screen (SOGS) [8–10]. The SOGS is a self-administered tool that evaluates gambling activity based on frequency, amount spent, problem occurrence, self-assessment, and sense of control. It allows for convenient scoring and assessment across a broad range of problem areas [9,11]. The second tool is the Problem Gambling Severity Index (PGSI) [12,13]. The PGSI evaluates the severity of gambling problems, including frequency of participation, financial impact, loss of control, and effects on daily life, and has high reliability and validity [12].

**Lines 102-105:**

The SOGS, PGSI, and DSM-5 are described above. LieBet, an early screening tool, consists of two questions focusing on lying and chasing behaviors related to gambling. While it can be administered quickly, it must be used alongside other tools for a comprehensive assessment [27].

**Comment:H**

Line(s) 137: It's common practice to amend this to 12 month or 6 months. Was there a specific reason why this was not done? You mention in the discussion that this is a limitation but some discussion in the method would be useful as well.

**Response:**

Thank you for your observation. The time frame for the SOGS was based on the Japanese government's survey of actual conditions. This has been clarified in the Materials and Methods section.

**Revised text**

**Line 105-108:**

The time frames for each screening test were adopted from their original versions: SOGS (Lifetime), PGSI, LieBet, and DSM-5 (12 months). Additionally, the Japanese government's survey on gambling conditions used the Lifetime time frame for the SOGS, which was adopted for this study.

**Comment:I**

Line(s) 152: This is new to me and is a rather elegant way of presenting this kind of data. However, the figure is low resolution making it harder to read the smaller scores.

**Response:**

While the resolution of the PDF for peer review is low, the original figures and tables submitted to PLOS ONE have high resolution. We will request assistance from the editorial department regarding this matter. Additionally, we have provided a link below for downloading a high-resolution version.

URL for download: <https://www.dropbox.com/scl/fi/pns1n26bl5832o5gh66nj/Figure_1_noda.pdf?rlkey=vfrzaen1b6kchu5b8juemvz2s&st=c47p3i3n&dl=0>

**Comment:J**

Line(s) 167-170: There has recently been a few systematic reviews that have examined this very topic, these should be examined and discussed:

<https://doi.org/10.1007/s10389-021-01678-9>

<https://doi.org/10.1186/s13722-021-00243-9>

<https://doi.org/10.1016/j.jclinepi.2019.12.022>

<https://doi.org/10.1016/j.cpr.2019.101784>

**Response:**

We appreciate your guidance. Your recommendations regarding these systematic reviews have been incorporated, and the relevant literature has been cited.

**Revised text:**

**Line 171-173:**

yet few researchers have focused on this issue, and survey design and implementation have received little attention in this regard [29].

**Comment:K**

Line(s) 188- 198: This is rather old, see below:

<https://doi.org/10.1007/s10389-021-01678-9>

<https://doi.org/10.1177/1073191113500522>

**Response:**

Thank you for your comment. We have replaced the reference.

**Revised text:**

**Line199-200:**

These results are consistent with those of prior research, which reports more false positives for the SOGS than for other screening tests [29].

**Comments from Reviewer #2:**

**Comment:L**

This study aims to investigate the relationship between scores on two gambling problem screening instruments and symptoms of gambling addiction. While efforts to improve the sensitivity of these instruments are commendable, this study would be strengthened by a more robust theoretical foundation and clearer methodological explanations.

**Response:**

Thank you for your valuable comments. We have responded to your points as described below, and have also revised the main manuscript. In line with the in-depth discussions during this revision, we have added one author and redefined the authors' roles.

**Comment:M**

Although the SOGS and PGSI has been traditionally used for screening, are increasingly employed to assess the severity of problems beyond just addiction symptoms. The authors should elaborate on this growing application and clearly define the types of behaviors, problems or symptoms assessed in each instrument, including DSM-5 criteria.

**Response:**

Thank you for your comment. We have added a detailed explanation of screening tests to the Introduction and Materials & methods sections, considering the opinion of reviewer 1 (comment G and P).

**Revised text:**

**Lines 68-74:**

The first of these is the South Oaks Gambling Screen (SOGS) [8–10]. The SOGS is a self-administered tool that evaluates gambling activity based on frequency, amount spent, problem occurrence, self-assessment, and sense of control. It allows for convenient scoring and assessment across a broad range of problem areas [9,11]. The second tool is the Problem Gambling Severity Index (PGSI) [12,13]. The PGSI evaluates the severity of gambling problems, including frequency of participation, financial impact, loss of control, and effects on daily life, and has high reliability and validity [12].

**Lines 102-105:**

The SOGS, PGSI, and DSM-5 are described above. LieBet, an early screening tool, consists of two questions focusing on lying and chasing behaviors related to gambling. While it can be administered quickly, it must be used alongside other tools for a comprehensive assessment [27].

**Comment:N**

Crucially, this study may be limited by a potential order effect, where the order in which the instruments were administered may influence how the data is interpreted. The limited description of the assessment procedure, lacking key details, makes it impossible to determine whether an order effect might have influenced the results. Administering three instruments with very similar questions, without counterbalancing the order, could bias participants' responses. This ambiguity regarding the order effect raises concerns about the trustworthiness of the study's findings.

**Response:**

Thank you for your observation. We agree with your suggestion and have added a note in the Limitations section regarding the order effect of the screening tests. This will also inform our approach in future research.

**Revised text:**

**Line 237-239:**

The fourth limitation is that the order of the four screening tests was not randomized. The order of administration may have influenced participant responses.

**Comment:O**

The study design seems to have originally aimed at tracking gambling prevalence in various Japanese prefectures before and after casinos opened. However, the link between this objective and the chosen measures and test analyses remains unclear. To improve reader understanding, the authors should explain this connection more clearly and provide additional context about gambling practices in Japan.

**Response:**

Thank you for your observationThe scales employed in this study had already been used in a large-scale survey in Japan, and the results were comparable. Thus, they were deemed appropriate for this study. Initially, we sought to measure the impact of the choice of screening method and cut-off score, as well as to compare prevalence before and after casino openings . However, after carefully considering the reviewers' comments, we recognized that the discussion on casino-related comparisons was underdeveloped and potentially distracting. Consequently, we have removed these elements from the study’s objectives.

**Deleted text:**

[At the end of Introduction]

In Japan, casinos were legalized in 2016 and are scheduled to open in the near future. Public concerns have been raised that establishing integrated resorts, including casinos, increases the prevalence of gambling addiction [21,22]. Several studies have also shown that the prevalence of gambling disorder increases after casinos open [4,23,24]. The secondary aim of our study is to conduct a baseline study in the period before the first casino opens in Japan.

[At the beginning of Materials and methods]

To provide a before-and-after comparison of the effect of opening casinos on the prevalence of possible gambling disorders,

**Comment:P**

Minor: A section on measures should be included, explaining all measures and their psychometric properties. Additionally, the section describing the sample and its recruitment procedure should be expanded.

**Response:**

Thank you for your comment. We have responded to this issue. Please see comment M.

**Comment:Q**

If one of the study's aims is to measure prevalence, then why not use a representative sample? Otherwise, I'm not convinced that the study can be used to make arguments about the effect of gambling availability.

**Response:**

Thank you for your observation.

We agree with your opinion. However, there are few national representative surveys on gambling disorders in Japan is limited, and data are often legally inaccessible for research purposes. As noted in studies like Mori et al., commercial databases are frequently employed in prevalence studies. This limitation highlights a key area for future research.
